# Association between polyunsaturated fatty acids intake and serum neurofilament light chain concentrations in American adults: a cross-sectional study

**DOI:** 10.3389/fnut.2025.1608211

**Published:** 2025-07-11

**Authors:** Yuxuan Wang, Jingyi Wang, Lifeng Wang

**Affiliations:** ^1^Department of Ophthalmology, The Affiliated Hospital of Qingdao University, Qingdao, Shandong, China; ^2^School of Basic Medicine, Qingdao Medical College, Qingdao, Shandong, China; ^3^Department of the Colorectal Anal Surgery, The Affiliated Taian City Central Hospital of Qingdao University, Taian, Shandong, China

**Keywords:** nutrition, polyunsaturated fatty acids, neurofilament light chain, epidemiology, public health

## Abstract

**Background:**

Serum neurofilament light chain (sNfL) is a promising blood-based biomarker for detecting neuroaxonal injury, with elevated levels observed in various neurological disorders. While polyunsaturated fatty acids (PUFAs) have been linked to favorable neurological outcomes, the relationship between dietary PUFAs intake and sNfL levels remains unclear. This study aimed to investigate the association between PUFAs intake and sNfL levels in American adults.

**Methods:**

A cross-sectional study was conducted using data from the National Health and Nutrition Examination Survey (NHANES) 2013–2014. Multivariable regression analyses were applied to examine the associations between individual PUFA, omega-3 PUFAs, omega-6 PUFAs, and omega-6/omega-3 ratio and sNfL levels. Restricted cubic spline (RCS) models were used to assess potential non-linear relationships. The overall effect of PUFAs mixtures on sNfL was assessed using quantile g-computation (QGC), while weighted quantile sum (WQS) regression was applied for sensitivity analysis.

**Results:**

A total of 1,109 eligible participants were included in the study. Alpha-linolenic acid (ALA), linoleic acid (LA), docosahexaenoic acid (DHA), docosapentaenoic acid (DPA), and eicosapentaenoic acid (EPA) were inversely associated with sNfL levels after adjusting for all covariates. Omega-3 and omega-6 PUFAs were negatively associated with sNfL, whereas the omega-6/omega-3 ratio was positively associated with sNfL. Findings from WQS and QGC analyses further supported an inverse association between PUFA mixtures and sNfL levels.

**Conclusion:**

This study indicates that PUFAs intake is associated with decreased levels of sNfL, suggesting a potential association with reduced neuroaxonal injury. Further studies are needed to validate these findings and explore the biological pathways.

## Introduction

Neurofilament light chain (NfL) is a neuron-specific cytoskeletal protein predominantly found in myelinated axons (1). Studies have shown that when axons are damaged or neurons degenerate, NfL is released from cells into extracellular fluid and eventually enters the blood circulation. Studies have shown that when nerve damage or neuronal degeneration occurs, NfL fragments are released into the extracellular fluid and enter the blood and cerebrospinal fluid (CSF) (2, 3). In recent years, serum neurofilament light chain (sNfL) has become an important biomarker for the assessment of nerve injury and neurodegenerative diseases (4, 5). The increased levels of sNfL have been observed to be associated with an elevated risk of onset of a variety of nervous system diseases, including multiple sclerosis (MS), Parkinson's disease (PD), Alzheimer's disease (AD), and traumatic brain injury (6–9). In addition, sNfL is thought to be involved in the accumulation of neuronal damage during the normal aging process, and its levels can increase with age (10). Despite the importance of sNfL in the study of neurodegenerative diseases and nerve injury, few studies have explored the environmental and dietary factors that influence its levels.

Dietary factors play an important role in human health (11, 12). Polyunsaturated fatty acids (PUFAs) are essential fatty acids in the body, which have physiological functions such as maintaining the structure of cell membranes, regulating neurotransmitters, and inflammatory responses (13). PUFAs can be classified into omega-3 and omega-6 PUFAs according to their carbon chain structure and double bond position (14). The consumption of omega-6 PUFAs, which are primarily derived from vegetable oils, seeds, and meat, has been demonstrated to decrease low-density lipoprotein cholesterol levels in the blood, thereby contributing to a reduction in cardiovascular mortality (15, 16). The primary sources of omega-3 PUFAs include deep-sea fish, nuts, and algae (17). Omega-3 polyunsaturated fatty acids have many health benefits, including enhanced visual and brain development in the fetus, and are associated with reduced risk of rheumatoid arthritis, obesity, diabetes, metabolic syndrome, cardiovascular disease, and other diseases (18–21). In addition, omega-3 PUFAs have been demonstrated to possess anti-inflammatory and anti-oxidative properties, with the capacity to modulate the synthesis and release of pro-inflammatory mediators, thereby contributing to the inhibition of neuroinflammation (22). Furthermore, maintaining an appropriate balance between omega-6 and omega-3 PUFAs intake is important for nervous system health (23).

Dietary factors play an important role in human health (12, 24). In recent years, an increasing number of studies have focused on the potential protective effects of PUFAs on the nervous system. Studies have shown that higher PUFAs intake may be associated with improved cognitive function and decreased risk of major depression, AD, and PD (25–27). However, although intake of PUFAs may protect neurons by reducing neuroinflammation and oxidative stress, the specific association between PUFAs and sNfL levels remains unclear. Current studies mainly focus on the effects of PUFAs on cognitive function, but there is still a lack of evidence on whether PUFAs can reduce neuronal axonal damage. Furthermore, the effects of PUFAs on sNfL may vary depending on individual characteristics (age, sex, ethnicity, etc.). Therefore, further investigation of the relationship between PUFAs intake and sNfL levels may provide new epidemiological evidence for the potential association with reduced neuroaxonal injury.

To comprehensively evaluate the relationship between individual and combined PUFA intake and sNfL levels, we conducted a cross-sectional analysis based on data from the National Health and Nutrition Examination Survey (NHANES), a large and nationally representative dataset. The analysis was rigorously adjusted for a wide range of confounding factors, including demographic variables and health-related characteristics, to enhance the validity and reliability of the findings.

## Methods

### Study population

**Figure 1:**
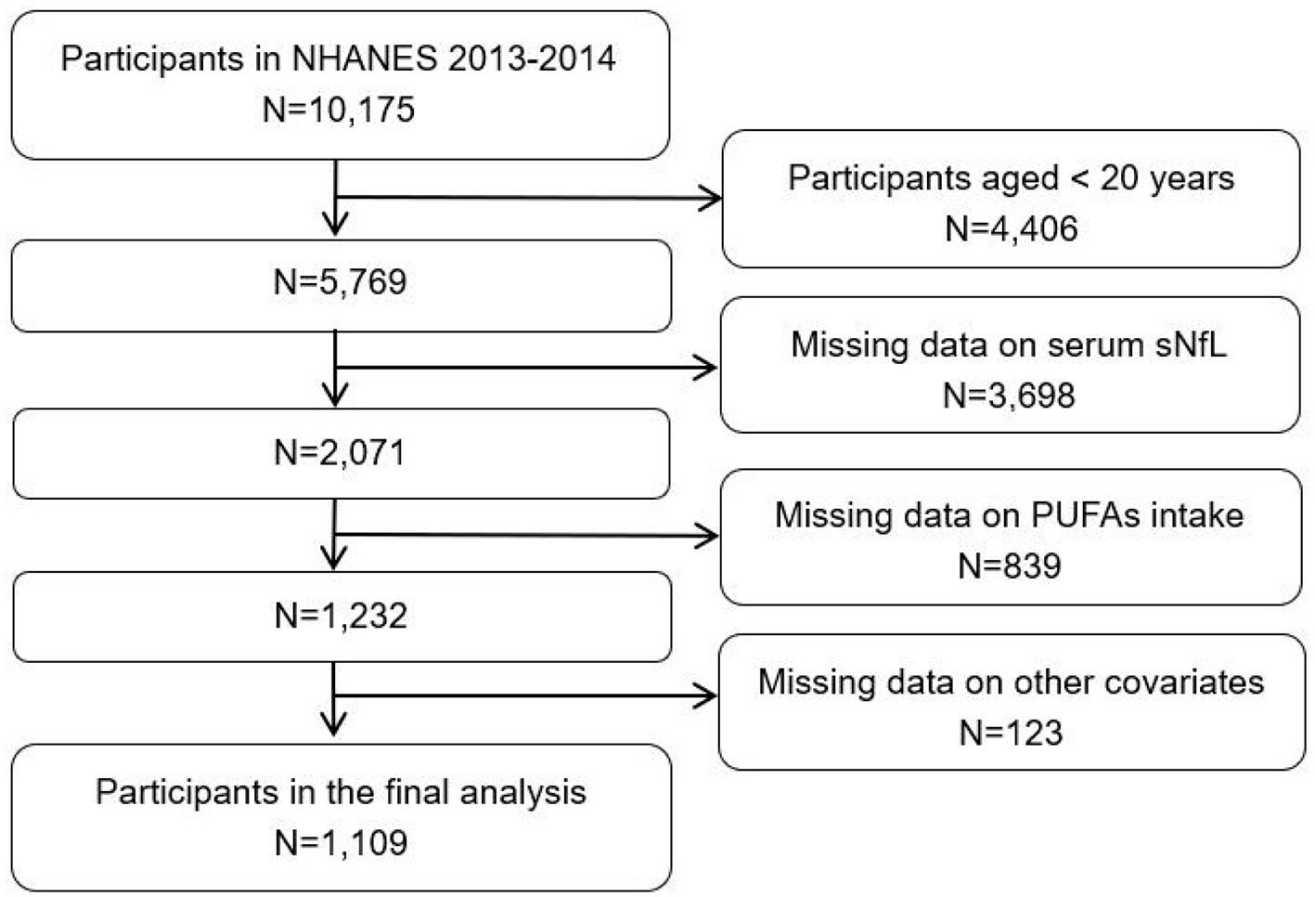
Flowchart of inclusion criteria for NHANES study participants (2005–2016).

This study extracted data spanning one NHANES cycle. A total of 10,175 participants were initially considered from the 2013–2014 NHANES cohort. The following are the exclusion criteria on participants: (1) non-adult participants (< 20 years, *N* = 4,406); (2) missing data on sNfL (*N* = 3,698); (3) missing data on dietary PUFAs intake (*N* = 839); (4) missing data on other variates including sex, age, race, education level, ratio of family income to poverty (PIR), body mass index (BMI), alcohol drinking, smoking status, blood high-density lipoprotein cholesterol (HDL-C), blood total cholesterol (TC), serum creatinine (Scr), energy intake, protein intake, diabetes, and cancer (*N* = 123). Finally, a total of 1,109 participants were enrolled in the study (Figure 1).

### Assessment of PUFAs intake

The estimation of PUFAs intake was conducted using dietary data. The assessment of dietary intake was undertaken through two non-consecutive 24-h dietary recall interviews, with the initial interview conducted in person and the subsequent interview conducted via telephone after 3–10 days. Nutrient intakes were derived using the American Department of Agriculture's Food and Nutrient Database for Dietary Studies (FNDDS), which converts reported food and beverage consumption into nutrient values, including PUFAs intake.

A total of seven PUFAs were included in this study, including linoleic acid (LA, 18:2), α-linoleic acid (ALA, 18:3), stearidonic acid (SDA, 18:4), arachidonic acid (AA, 20:4), eicosapentaenoic acid (EPA, 20:5), docosapentaenoic acid (DPA, 22:5), and docosahexaenoic acid (DHA, 22:6). According to the classification of previous studies, omega-3 PUFAs consists of five subtypes: ALA, SDA, EPA, DPA, and DHA, while omega-6 PUFAs consists of two subtypes: LA and AA (28). The total polyunsaturated fatty acid (TPFA) was the sum of seven PUFAs. The average intake from the two dietary recall days was calculated and used in the statistical analyses.

### Measurement of sNfL

Serum neurofilament light chain (sNfL) levels were measured using a high-sensitivity chemiluminescence immunoassay (Siemens Healthineers) on the fully automated Atellica system. The assay employs acridinium ester-labeled antibodies and paramagnetic particles for antigen detection, with signal quantification based on chemiluminescence. All measurements were conducted following standardized quality control procedures, including routine analysis of low, medium, and high concentration QC samples to ensure accuracy and reliability. Levels of sNfL were measured from fasting blood samples collected at the Mobile Examination Center (MEC) during the participants' in-person visit, coinciding with the first 24-h dietary recall.

### Covariates

The selection of covariates was based on prior literature and biological plausibility. Variables that may influence both PUFA intake and sNfL levels, including demographics, socioeconomic status, lifestyle behaviors, metabolic factors, and comorbidities (e.g., diabetes, cancer), were included to control for potential confounding. Demographic variables included sex (male or female), age group (< 50 or ≥50 years), race/ethnicity (Mexican American, other Hispanic, non-Hispanic White, non-Hispanic Black, and other racial groups), educational attainment (less than high school, high school graduate, and college graduate), and poverty income ratio (PIR), categorized as < 1, 1–3, and >3. Additional covariates included body mass index (BMI, kg/m^2^), alcohol consumption (yes, no), smoking status (yes, no), blood levels of high-density lipoprotein cholesterol (HDL-C, mg/dl) and total cholesterol (TC, mg/dl), estimated glomerular filtration rate (eGFR, ml/min), total energy intake (kcal), protein intake (g), as well as diabetes status (yes, no, or borderline), and cancer (yes, no). Estimated glomerular filtration rate was calculated using the CKD-EPI 2009 equation based on age, sex, race, and Scr levels (29).

### Statistical analyses

Continuous variables were summarized as means with standard deviations (mean ± SD), whereas categorical variables were reported as frequencies and corresponding percentages. Group comparisons were performed using independent *t*-tests for continuous variables and chi-square tests for categorical variables. All statistical analyses incorporated the NHANES complex sampling design. Specifically, the serum neurofilament light chain 2-year subsample weights (WTSSNH2Y), along with the corresponding strata and primary sampling units, were applied to account for unequal probability of selection and to produce nationally representative estimates.

Both serum neurofilament light chain concentrations and PUFA intake were log_10−_transformed prior to analysis to improve normality and reduce skewness. Weighted multivariable regression analyses were employed to assess the association between PUFA intake and sNfL levels, yielding beta coefficients with corresponding 95% confidence intervals (CIs). Three models were constructed: model 1 was unadjusted; model 2 accounted for sex, age, race, and BMI; and model 3 further adjusted for all covariates. A restricted cubic spline (RCS) regression was performed to explore potential non-linear relationships between PUFA intake and sNfL levels (30). To evaluate the overall association between multiple PUFA exposures and sNfL, two statistical approaches were applied: quantile G-computation (QGC) and weighted quantile sum (WQS) regression (31, 32). These advanced models allow for the assessment of joint effects in exposure mixtures. Stratified analyses were conducted based on sex and age (< 50 vs. ≥50 years). In all analyses, PUFA intake served as the exposure variable, and sNfL concentration was treated as the outcome.

All statistical analyses were performed using R software and EmpowerStats.

## Results

### Baseline characteristics

**Figure 2:**
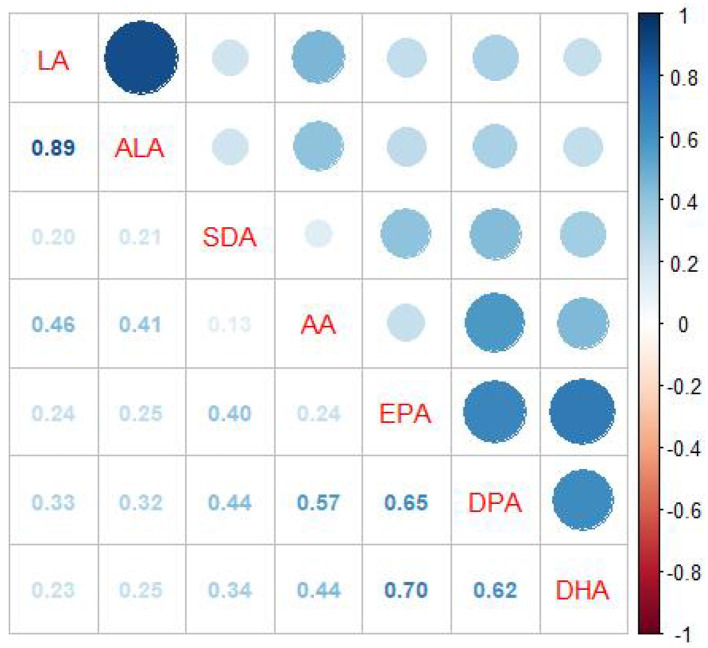
Spearman correlation coefficient plot. Correlation coefficients (range: −1 to 1) are visualized by color intensity, indicating the strength of associations.

This study encompassed 1,109 participants (47.52% male and 52.48% female) with an average age of 46.32 ± 15.19 years. Participants were grouped according to the concentration of sNfL. There were statistical differences between the three groups in terms of sex, age, BMI, race, PIR, Scr, eGFR, smoking status, diabetes, cancer, DHA, and omega-6/omega-3 ratio but not in terms of education level, TC, HDL-C, energy intake, protein intake, alcohol drinking, TPFA, omega-6 PUFAs, omega-3 PUFAs, LA, AA, ALA, SDA, EPA, and DPA (Table 1). The overall correlation between PUFAs intake was positive (Figure 2).

**Table 1:** Baseline characteristics of participants from NHANES 2013–2014.

**Variables**	**Serum neurofilament light chain (pg/ml)**			***P*-value**
	**T1 (0.447–0.964)** ***N*** = **369**	**T2 (0.964–1.190)** ***N*** = **368**	**T3 (1.190–2.697)** ***N*** = **372**	
**Sex (%)**				**0.023**
Male	160 (43.48%)	195 (52.94%)	191 (51.28%)	
Female	209 (56.52%)	173 (47.06%)	181 (48.72%)	
Age (years)	35.37 ± 11.11	46.08 ± 13.82	54.02 ± 13.90	**< 0.001**
**Race (%)**				**< 0.001**
Mexican American	50 (13.54%)	24 (6.39%)	21 (5.59%)	
Other Hispanic	21 (5.79%)	22 (6.01%)	14 (3.70%)	
Non-Hispanic White	210 (56.79%)	250 (67.93%)	279 (75.13%)	
Non-Hispanic Black	61 (16.59%)	44 (12.02%)	40 (10.69%)	
Other race	27 (7.29%)	28 (7.65%)	18 (4.89%)	
**Education level (%)**				0.350
Under high school	62 (16.70%)	51 (13.90%)	52 (14.05%)	
High school	78 (21.16%)	66 (17.94%)	84 (22.60%)	
College graduate	229 (62.14%)	251 (68.16%)	236 (63.35%)	
PIR	2.74 ± 1.62	3.07 ± 1.73	3.13 ± 1.67	**0.003**
BMI (kg/m^2^)	30.44 ± 8.03	29.08 ± 7.32	30.69 ± 8.23	**0.012**
TC (mg/dL)	187.03 ± 41.42	188.66 ± 38.56	191.93 ± 41.72	0.250
HDL (mg/dL)	52.44 ± 13.63	53.18 ± 14.96	55.08 ± 17.95	0.061
Scr (mg/dL)	0.81 ± 0.17	0.87 ± 0.18	0.94 ± 0.30	**< 0.001**
**eGFR (ml/min)**				**< 0.001**
< 60	1 (0.32%)	11 (3.08%)	32 (8.69%)	
≥60	368 (99.68%)	357 (96.92%)	340 (91.31%)	
Energy (kcal)	2,238.50 ± 759.10	2,214.00 ± 796.47	2,251.73 ± 850.31	0.811
Protein (gm)	88.30 ± 31.05	88.81 ± 34.08	88.84 ± 37.42	0.972
**Alcohol drinking (%)**				0.116
Yes	293 (79.51%)	300 (81.59%)	281 (75.46%)	
No	76 (20.49%)	69 (18.41%)	91 (24.54%)	
**Smoking status (%)**				**0.002**
Yes	134 (36.37%)	173 (46.93%)	180 (48.26%)	
No	235 (63.63%)	195 (53.07%)	192 (51.74%)	
**Diabetes (%)**				**< 0.001**
Yes	13 (3.45%)	25 (6.96%)	68 (18.24%)	
No	342 (92.61%)	331 (89.90%)	293 (78.73%)	
Borderline	14 (3.94%)	12 (3.14%)	11 (3.03%)	
**Cancer (%)**				**< 0.001**
Yes	21 (5.63%)	33 (9.10%)	58 (15.65%)	
No	348 (94.37%)	335 (90.90%)	314 (84.35%)	
**Fatty acids (mg)**			
TPFA	4.28 ± 0.20	4.25 ± 0.21	4.27 ± 0.21	0.153
Omega-6	4.22 ± 0.21	4.19 ± 0.21	4.22 ± 0.21	0.136
LA (C18:2)	4.22 ± 0.21	4.19 ± 0.21	4.21 ± 0.21	0.134
AA (C20:4)	2.17 ± 0.23	2.16 ± 0.28	2.15 ± 0.29	0.788
Omega-3	3.28 ± 0.22	3.24 ± 0.22	3.25 ± 0.23	0.106
ALA (C18:3)	3.24 ± 0.22	3.20 ± 0.22	3.21 ± 0.23	0.126
SDA (C18:4)	0.57 ± 0.72	0.61 ± 0.71	0.51 ± 0.69	0.194
EPA (C20:5)	1.18 ± 0.53	1.18 ± 0.57	1.13 ± 0.55	0.425
DPA (C22:5)	1.35 ± 0.35	1.34 ± 0.34	1.31 ± 0.34	0.157
DHA (C22:6)	1.45 ± 0.68	1.50 ± 0.71	1.35 ± 0.71	**0.015**
Omega6/Omega3	9.19 ± 2.34	9.31 ± 2.69	9.66 ± 2.63	**0.038**

Values in this table were represented as mean ± SD or number (%). PUFAs intake and sNfL levels were log-transformed using base 10 logarithm. Bold values indicate statistical significance at *P* < 0.05.

### Association between PUFAs intake and sNfL

**Figure 3:**
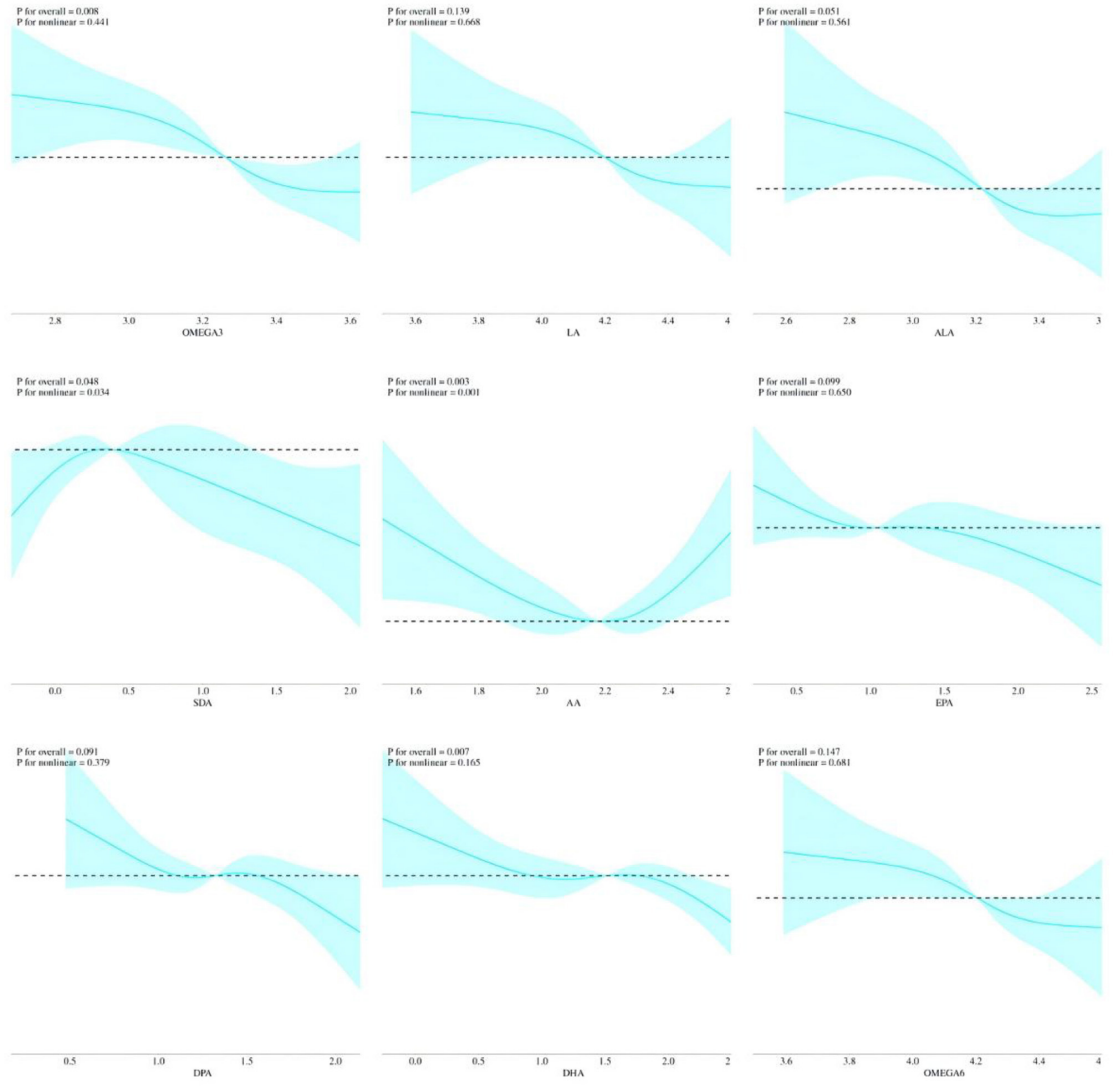
Non-linear relationships between PUFAs intake and sNfL by RCS. All covariates were adjusted in the RCS model.

The results showed that intake of TPFA (β = −0.131, 95% CI: −0.228, −0.035), omega-6 PUFAs (β = −0.117, 95% CI: −0.213, −0.022), LA (β = −0.118, 95% CI: −0.213, −0.023), omega-3 PUFAs (β = −0.172, 95% CI: −0.252, −0.091), ALA (β = −0.134, 95% CI: −0.215, −0.054), EPA (β = −0.046, 95% CI: −0.074, −0.018), DPA (β = −0.059, 95% CI: −0.110, −0.009), and DHA (β = −0.051, 95% CI: −0.073, −0.028) were negatively associated with sNfL after adjusting for all covariates. The omega-6/omega-3 ratio was positively associated sNfL (β = 0.010, 95% CI: 0.004, 0.016). However, the association between AA and SDA and sNfL was not statistically significant (Table 2). As a sensitivity analysis, we reanalyzed the data by including participants with complete information on PUFA intake, serum sNfL, and core covariates (sex, age, race, and BMI), resulting in a sample size of 1,246 individuals. The weighted multivariable regression analyses yielded results consistent with the main findings, supporting the robustness of the observed associations (Supplementary Table S1). RCS models were employed to further investigate potential non-linear associations between PUFA intake and sNfL levels. The analysis revealed distinct threshold effects for certain PUFAs, notably SDA and AA (Figure 3).

**Table 2:** Associations of PUFAs intake with sNfL.

**Exposure**	**Model 1**	**Model 2**	**Model 3**
	β **(95% CI)** ***P*****-value**	β **(95% CI)** ***P*****-value**	β **(95% CI)** ***P*****-value**
TPFA	−0.051 (−0.132, 0.030) 0.219	−0.068 (−0.140, 0.004) 0.063	−0.131 (−0.228, −0.035) **0.008**
Omega-6	−0.047 (−0.127, 0.033) 0.249	−0.060 (−0.131, 0.010) 0.095	−0.117 (−0.213, −0.022) **0.016**
LA (C18:2)	−0.047 (−0.127, 0.032) 0.243	−0.061 (−0.131, 0.010) 0.092	−0.118 (−0.213, −0.023) **0.015**
AA (C20:4)	−0.012 (−0.075, 0.051) 0.709	−0.003 (−0.059, 0.054) 0.925	−0.006 (−0.074, 0.062) 0.863
Omega-3	−0.077 (−0.151, −0.003) **0.042**	−0.112 (−0.177, −0.047) ** < 0.001**	−0.172 (−0.252, −0.091) ** < 0.001**
ALA (C18:3)	−0.067 (−0.141, 0.007) 0.078	−0.084 (−0.149, −0.019) **0.012**	−0.134 (−0.215, −0.054) **0.001**
SDA (C18:4)	−0.003 (−0.027, 0.020) 0.776	−0.012 (−0.032, 0.009) 0.263	−0.013 (−0.034, 0.008) 0.227
EPA (C20:5)	−0.025 (−0.056, 0.005) 0.105	−0.046 (−0.073, −0.020) ** < 0.001**	−0.046 (−0.074, −0.018) **0.002**
DPA (C22:5)	−0.052 (−0.101, −0.003) **0.036**	−0.057 (−0.100, −0.014) **0.009**	−0.059 (−0.110, −0.009) **0.021**
DHA (C22:6)	−0.022 (−0.046, 0.002) 0.068	−0.047 (−0.067, −0.026) ** < 0.001**	−0.051 (−0.073, −0.028) ** < 0.001**
Omega6/Omega3	0.007 (0.000, 0.013) **0.040**	0.010 (0.004, 0.016) ** < 0.001**	0.010 (0.004, 0.016) ** < 0.001**

Multivariable regression models assessing the associations between PUFAs intake and sNfL. Bold values indicate statistical significance at *P* < 0.05.

### Association between mixed PUFAs intake with blood lipids

**Figure 4:**
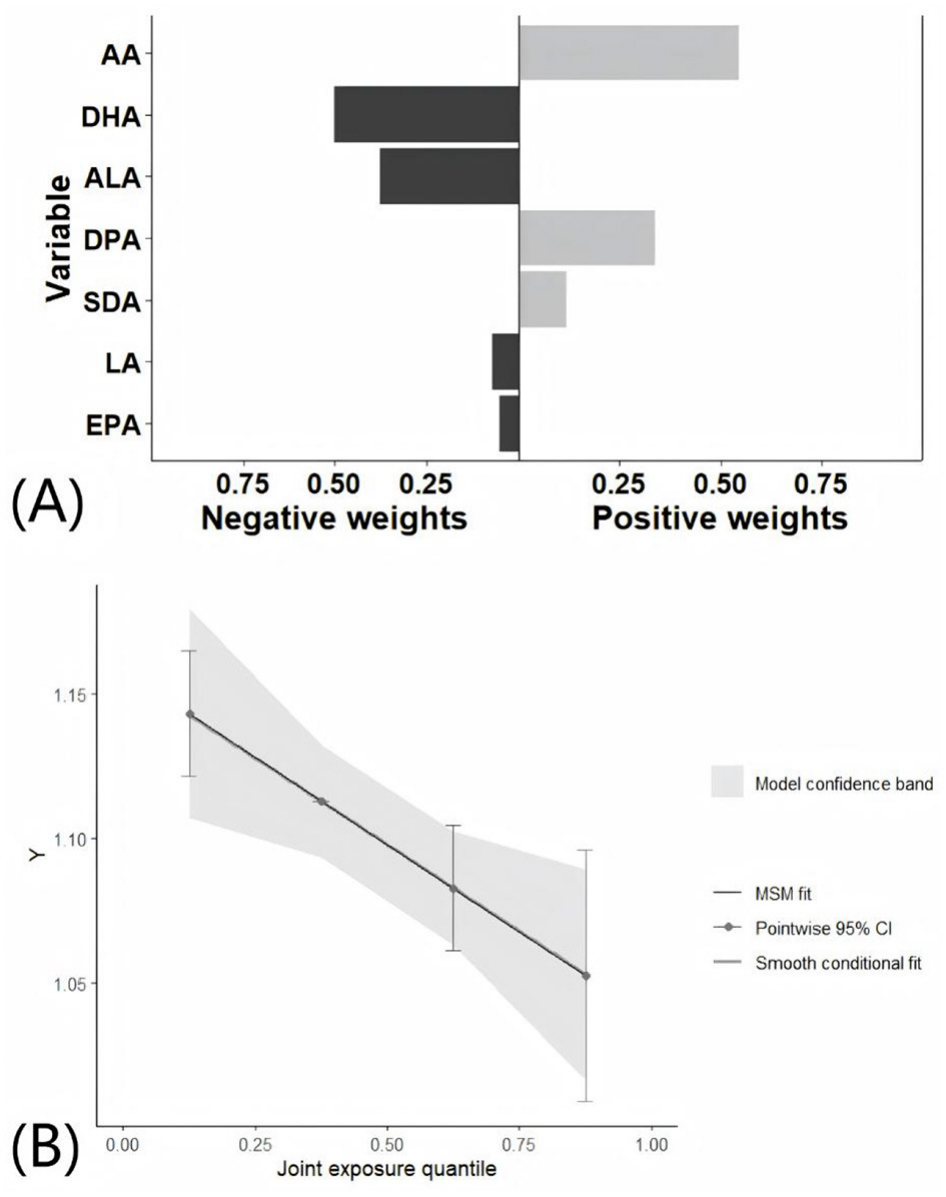
Association between mixed PUFAs intake with sNfL by QGC analyses. All covariates were adjusted in the QGC model. **(A)** Effects of individual PUFA intake on sNfL by QGC analyses. **(B)** Overall effect of mixed PUFAs intake on sNfL by QGC analyses.

The main results of the mixed effect by WQS regression were consistent with QGC analyses. QGC analyses suggested that mixed PUFAs intake were negatively associated with sNfL (β = −0.030, 95% CI: −0.052, −0.008). AA contributed most on the positive weights, while DHA contributed most on the negative weights (Figure 4A). Using the lowest quartile of the PUFA mixture as the reference group, a significant decreasing trend in sNfL levels was observed with increasing concentrations of the mixture (Figure 4B). Results from the WQS regression indicated a negative association between mixed PUFA intake and sNfL levels after adjustment for all covariates (β = −0.026, 95% CI: −0.050, −0.002). Furthermore, WQS analysis identified AA as the predominant contributor to the overall mixture effect, accounting for 71.5% of the weight (Supplementary Figure S1A). Scatter plot showed that PUFAs intake were negatively correlated with sNfL (Supplementary Figure S1B).

### Stratified analyses by sex and age

**Figure 5:**
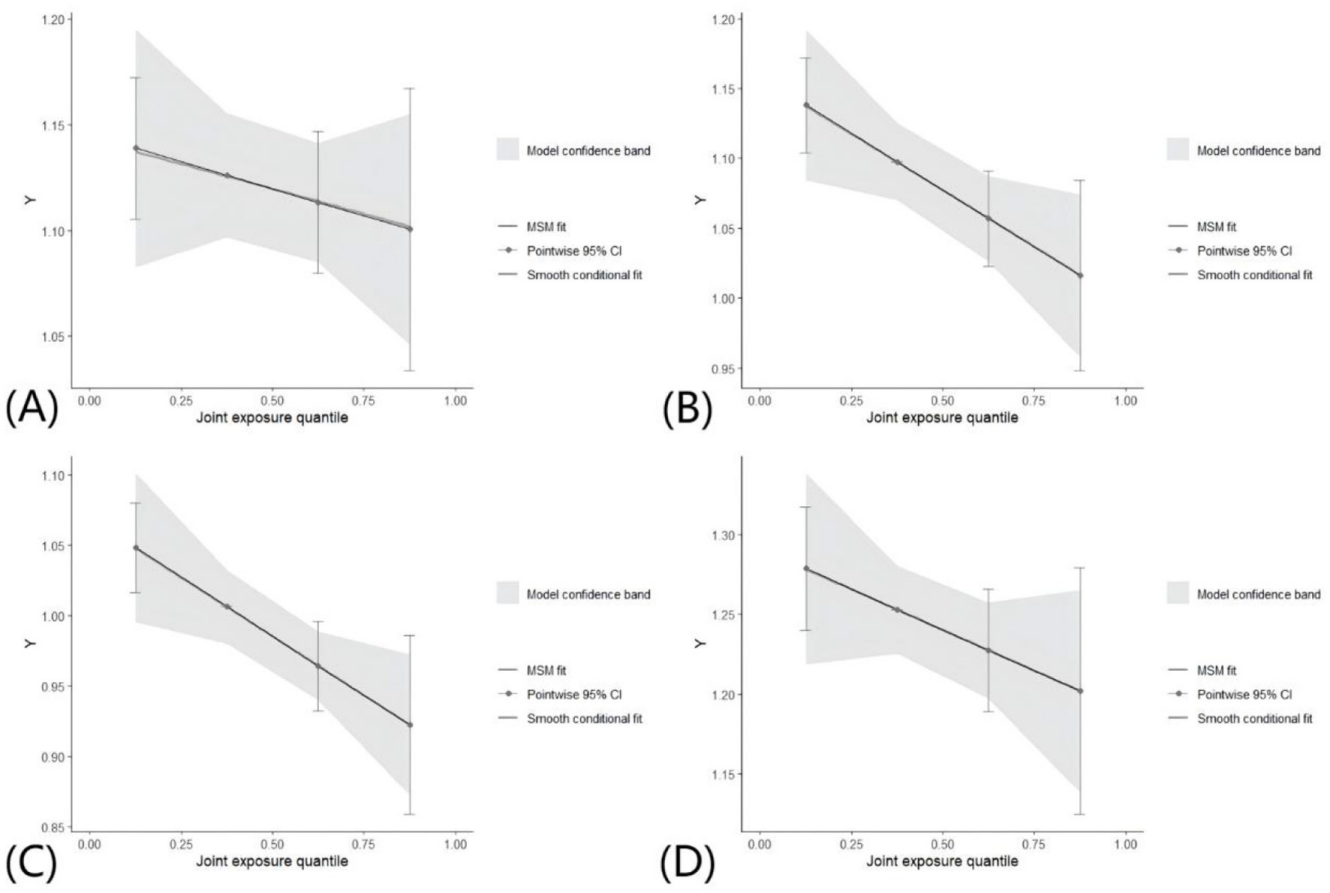
Association between mixed PUFAs intake with sNfL by QGC analyses in different groups. All covariates were adjusted in the QGC model. **(A)** Overall effect of mixed PUFAs intake on sNfL in males. **(B)** Overall effect of mixed PUFAs intake on sNfL in females. **(C)** Overall effect of mixed PUFAs intake on sNfL in younger participants. **(D)** Overall effect of mixed PUFAs intake on sNfL in older participants.

The analysis included a total of 527 males and 582 females. Multivariable regression models demonstrated that the intake of omega-3 PUFAs, ALA, and DHA was negatively associated with sNfL in males, after adjusting for covariates. In contrast, the intake of TPFA, omega-6 PUFAs, omega-3 PUFAs, and four single PUFAs was negatively associated with sNfL in females. The omega-6/omega-3 ratio was positively associated sNfL in both male and female participants (Supplementary Table S2). The QGC model showed that mixed PUFAs intake was negatively associated with sNfL in females (β = −0.041, 95% CI: −0.075, 0.007, *P* = 0.019). However, no significant association was observed in males (β = −0.013, 95% CI: −0.046, 0.021, *P* = 0.454; Figures 5A, B).

Following the categorization of participants based on age, the sample was divided into two groups: 620 individuals younger than 50 years and 489 individuals older than or equal to 50 years. Multivariable regression models showed that TPFA, omega-6 PUFAs, omega-3 PUFAs, LA, ALA, EPA, DPA, and DHA exhibited a negative correlation with sNfL, after adjusting for covariates, in the younger participants. The omega-6/omega-3 ratio was positively associated sNfL. In contrast, only DHA was negatively associated with sNfL in the older participants (Supplementary Table S3). The QGC model revealed a negative association between mixed PUFAs intake and sNfL in younger participants (β = −0.042, 95% CI: −0.074, −0.010, *P* = 0.010), while no significant association was observed in older participants (β = −0.026, 95% CI: −0.064, 0.013, *P* = 0.195; Figures 5C, D).

## Discussion

This study investigated the association between PUFAs intake and sNfL levels in American adults using NHANES data. The results demonstrated that higher intake of TPFA, omega-6 PUFAs, LA, omega-3 PUFAs, ALA, EPA, DPA, and DHA was significantly associated with lower sNfL levels after adjusting for all covariates. Conversely, a higher omega-6/omega-3 ratio was positively associated with sNfL. Both the WQS and QGC models further supported an inverse association between mixed PUFAs intake and sNfL. Stratified analyses revealed that the strength of this association varied across subgroups. These findings suggest that PUFAs intake may contribute to lower sNfL levels, highlighting a potential association with reduced neuroaxonal injury.

Previous studies have investigated the effects of PUFAs supplementation on sNfL levels, particularly in neurodegenerative diseases and inflammatory conditions, yielding mixed results. Some findings contrast with those of the present study. A randomized controlled trial in AD patients (*n* = 33, treatment: *n* = 18, placebo: *n* = 15) reported that 6 months of daily supplementation with 2.3 g of omega-3 PUFAs led to a significant increase in CSF NfL levels (*P* < 0.05), potentially indicating heightened inflammatory activity and axonal damage, with no observed correlation to cognitive function (33). Similarly, in amyotrophic lateral sclerosis (ALS) patients, treatment with a deuterated linoleic acid derivative showed no significant effect on plasma NfL levels (34). Another study in American football athletes found that DHA+EPA supplementation, despite increasing plasma fatty acid levels, did not mitigate the rise in sNfL associated with repetitive head impacts (35).

Conversely, several studies align with our findings. A randomized, placebo-controlled trial in collegiate football players (*n* = 81) evaluating DHA supplementation at doses of 2, 4, and 6 g/day over a competitive season found that DHA intake increased plasma DHA in a dose-dependent manner and likely attenuated NfL elevations, suggesting a neuroprotective effect (36). Additionally, omega-3 supplementation in another cohort of football athletes was associated with lower sNfL levels compared to controls, reinforcing its potential role in neuroprotection (37). These findings suggest that PUFAs intake may contribute to lower sNfL levels, reinforcing its potential role in neuroprotection.

The observed inverse association between PUFAs intake and sNfL levels may be attributed to several biological mechanisms. First, PUFAs, particularly omega-3 fatty acids (DHA and EPA), are integral components of neuronal cell membranes and play a crucial role in maintaining axonal integrity and synaptic plasticity. DHA has been shown to enhance membrane fluidity, modulate ion channel function, and support neurotrophic signaling, which may help protect axons from degeneration (38, 39). Second, PUFAs exert strong anti-inflammatory effects by reducing the production of pro-inflammatory cytokines such as tumor necrosis factor-alpha and interleukin-6 while promoting the synthesis of specialized pro-resolving lipid mediators (40–42). These molecules have effects in resolving neuroinflammation and mitigating secondary damage to axons. In addition, PUFAs have antioxidant effects that may counteract oxidative stress-induced neurotoxicity, which is a key driver of axonal injury and neurofilament release (43, 44). Further studies are required to explore the precise mechanism.

Stratified analysis revealed that the inverse association between PUFAs intake and sNfL levels was significant in females, but not in males. Several potential biological mechanisms may underlie this sex-specific difference. Estrogen has been shown to enhance neuroprotection by increasing DPA and DHA uptake, promoting anti-inflammatory lipid mediator synthesis and modulating neurotrophic signaling (45, 46). These effects may amplify the beneficial impact of PUFAs intake on neuroaxonal integrity in females. Sex differences in PUFAs metabolism could also explain the observed results. Females generally exhibit higher DPA and EPA to DHA conversion rates compared to males (47). In addition, stratified analysis also revealed that the inverse association between PUFAs intake and sNfL levels was significant in individuals under 50 years old but not in those aged 50 and above. Several factors may explain this age-related difference. Younger individuals generally exhibit greater neuroplasticity and axonal repair capacity, allowing PUFAs to exert stronger neuroprotective effects by reducing inflammation, stabilizing axonal integrity, and mitigating oxidative stress. In contrast, older individuals may have accumulated chronic neurodegenerative changes, making dietary interventions less effective in modulating sNfL levels (48–50). Previous studies have shown that sNfL levels gradually increase with the process of aging (10, 51). The higher sNfL level in the elderly may make the decrease in sNfL level caused by PUFAs intake insignificant.

The overall impact of PUFA intake on sNfL levels was assessed using two complementary analytical approaches: WQS regression and QGC analysis. Both methods consistently demonstrated an inverse association between mixed PUFA intake and sNfL levels. However, the contribution of individual PUFA subtypes to the overall effect varied between the two techniques due to differences in their underlying statistical frameworks (31, 32).

The QGC analysis accommodates both interaction effects and non-linear relationships among exposures, thereby enhancing the ability to detect the influence of specific PUFAs when such complexities exist. In contrast, WQS regression utilizes a linear weighting scheme to summarize the effects of multiple exposures, which may limit its sensitivity to intricate patterns such as non-linearity or inter-exposure interactions. As a result, certain PUFA subtypes may exhibit greater apparent influence in QGC analysis due to its superior capacity to identify these complex associations that WQS regression might overlook. Furthermore, WQS assumes a unidirectional effect of all exposures on the outcome, favoring those whose impacts are consistent with the overall trend. When some PUFAs exert effects in opposing directions, WQS may downweight or omit them, potentially obscuring their individual contributions. In contrast, QGC permits each exposure to independently influence the outcome, offering a more detailed assessment of the distinct effects associated with each PUFA.

This study presents several notable strengths. First, it is the first large-scale cross-sectional analysis to systematically examine the association between PUFA intake and sNfL levels. Additionally, advanced analytical approaches—specifically, the WQS and QGC models—were employed to assess the combined effects of multiple PUFAs. Moreover, sensitivity analyses using an alternative sample with less stringent exclusion criteria yielded consistent results, further supporting the robustness of the findings.

This study also has some limitations. Due to the cross-sectional study design, it is only possible to demonstrate an association between PUFAs intake and sNfL, rather than a causal relationship. Another limitation is the potential for reverse causation, as higher sNfL levels may reflect underlying neurological conditions rather than dietary exposures. We were unable to fully exclude individuals with undiagnosed or subclinical neurological conditions. Furthermore, dietary PUFAs intake was assessed using two non-consecutive 24-h dietary recalls, which may be subject to within-person day-to-day variability within individuals and may not fully capture participants' habitual long-term intake. While using the average of two recalls reduces random error compared to a single-day recall, residual measurement error is still likely to occur, which could weaken the observed associations. A substantial proportion of participants were excluded due to missing data on variables, resulting in a final analytic sample representing ~11% of the initial eligible population. This may introduce selection bias, and the generalizability of our findings may be limited to individuals with complete data. Additionally, the data on sNfL is only available in the cycle of NHANES 2013–2014, which limits the completion of the analysis with the latest data.

## Conclusion

This study demonstrates that PUFAs intake is associated with decreased level of sNfL, suggesting a potential association with reduced neuroaxonal injury. These findings underscore the potential public health implications of PUFA intake and highlight the importance of further research to elucidate the underlying biological mechanisms and inform effective preventive strategies.

## Data Availability

The original contributions presented in the study are included in the article/Supplementary material, further inquiries can be directed to the corresponding author.
