# Supplementary material for: Association between polyunsaturated fatty acids intake and serum neurofilament light chain concentrations in American adults: a cross-sectional study
Source: Front Nutr. 2025 Jul 11;12:1608211. doi: 10.3389/fnut.2025.1608211 (PMC12289513; doi:10.3389/fnut.2025.1608211)
Supplement: Supplementary file 1 [file Data_Sheet_1.docx]

**Supplement File**

Figure S1. Association between mixed PUFAs intake with sNfL by WQS regression


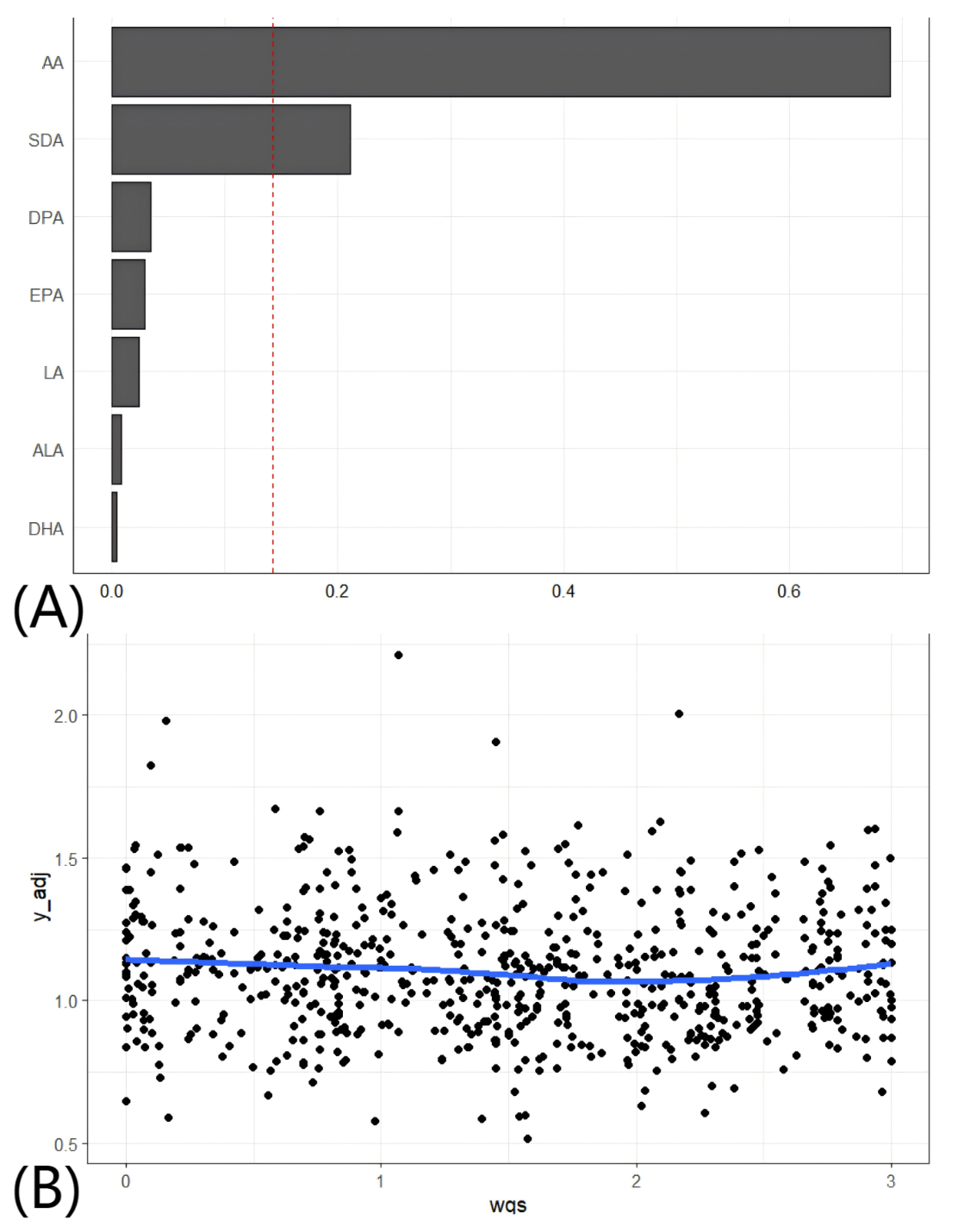


All covariates were adjusted in the WQS model. A: Proportion of effect of single PUFA intake on sNfL. B: The scatterplot of the overall effect of PUFAs intake on sNfL. Each black dot represents an individual observation, and the blue line indicates the trend.

Table S1. Sensitive analysis

| Exposure | Model 1 | Model 2 |
| --- | --- | --- |
|  | β (95 % CI) P-value | β (95 % CI) P-value |
| TPFA | -0.051 (-0.128, 0.026) 0.191 | -0.073 (-0.141, -0.005) **0.036** |
| Omega-6 | -0.056 (-0.132, 0.019) 0.144 | -0.073 (-0.140, -0.006) **0.034** |
| LA (C18:2) | -0.056 (-0.132, 0.019) 0.143 | -0.073 (-0.140, -0.006) **0.033** |
| AA (C20:4) | -0.034 (-0.093, 0.024) 0.251 | -0.024 (-0.077, 0.029) 0.372 |
| Omega-3 | -0.096 (-0.166, -0.026) **0.008** | -0.132 (-0.194, -0.070) **<0.001** |
| ALA (C18:3) | -0.087 (-0.157, -0.017) **0.015** | -0.106 (-0.168, -0.044) **<0.001** |
| SDA (C18:4) | -0.004 (-0.027, 0.019) 0.745 | -0.012 (-0.032, 0.008) 0.229 |
| EPA (C20:5) | -0.030 (-0.059, -0.001) **0.044** | -0.048 (-0.073, -0.022) **<0.001** |
| DPA (C22:5) | -0.053 (-0.099, -0.007) **0.025** | -0.057 (-0.098, -0.016) **0.007** |
| DHA (C22:6) | -0.025 (-0.047, -0.002) **0.031** | -0.049 (-0.069, -0.029) **<0.001** |
| Omega6/Omega3 | 0.009 (0.003, 0.015) **0.006** | 0.012 (0.006, 0.017) **<0.001** |

Model 1 was unadjusted; Model 2 accounted for gender, age, race, and BMI. (N=1,246) Bold values indicate statistical significance at P < 0.05.

Table S2. Stratified analysis by gender

| Exposure | Male | Female |
| --- | --- | --- |
|  | β (95 % CI), P-value | β (95 % CI), P-value |
| TPFA | -0.106 (-0.253, 0.041) 0.157 | -0.187 (-0.322, -0.053) **0.006** |
| Omega-6 | -0.091 (-0.234, 0.053) 0.216 | -0.173 (-0.306, -0.040) **0.011** |
| LA (C18:2) | -0.092 (-0.234, 0.051) 0.207 | -0.173 (-0.305, -0.041) **0.010** |
| AA (C20:4) | 0.011 (-0.091, 0.114) 0.831 | -0.025 (-0.120, 0.070) 0.608 |
| Omega-3 | -0.174 (-0.297, -0.051) **0.006** | -0.196 (-0.305, -0.087) **<0.001** |
| ALA (C18:3) | -0.134 (-0.258, -0.009) **0.036** | -0.163 (-0.271, -0.054) **0.003** |
| SDA (C18:4) | -0.016 (-0.047, 0.015) 0.305 | -0.014 (-0.044, 0.016) 0.374 |
| EPA (C20:5) | -0.022 (-0.063, 0.020) 0.306 | -0.066 (-0.106, -0.026) **0.001** |
| DPA (C22:5) | -0.071 (-0.144, 0.003) 0.060 | -0.043 (-0.116, 0.030) 0.249 |
| DHA (C22:6) | -0.052 (-0.084, -0.020) **0.002** | -0.048 (-0.080, -0.016) **0.003** |
| Omega6/Omega3 | 0.010 (0.002, 0.019) **0.013** | 0.010 (0.002, 0.018) **0.019** |

All covariates were adjusted in the multivariable regression model. P-values were calculated using weighted analyses. Bold values indicate statistical significance at P < 0.05.

Table S3. Stratified analysis by age

| Exposure | <50 years | ≥50 years |
| --- | --- | --- |
|  | β (95 % CI), P-value | β (95 % CI), P-value |
| TPFA | -0.164 (-0.303, -0.025) **0.021** | -0.095 (-0.236, 0.046) 0.187 |
| Omega-6 | -0.145 (-0.282, -0.008) **0.039** | -0.092 (-0.230, 0.046) 0.193 |
| LA (C18:2) | -0.146 (-0.282, -0.009) **0.037** | -0.092 (-0.229, 0.046) 0.191 |
| AA (C20:4) | -0.040 (-0.143, 0.063) 0.448 | -0.002 (-0.096, 0.092) 0.967 |
| Omega-3 | -0.248 (-0.363, -0.133) **<0.001** | -0.056 (-0.173, 0.062) 0.352 |
| ALA (C18:3) | -0.223 (-0.338, -0.107) **<0.001** | -0.022 (-0.139, 0.096) 0.717 |
| SDA (C18:4) | -0.000 (-0.032, 0.031) 0.981 | -0.013 (-0.043, 0.017) 0.404 |
| EPA (C20:5) | -0.054 (-0.098, -0.010) **0.017** | -0.032 (-0.071, 0.006) 0.098 |
| DPA (C22:5) | -0.084 (-0.159, -0.009) **0.028** | -0.054 (-0.125, 0.017) 0.136 |
| DHA (C22:6) | -0.050 (-0.083, -0.016) **0.004** | -0.047 (-0.077, -0.016) **0.003** |
| Omega6/Omega3 | 0.017 (0.009, 0.026) **<0.001** | -0.002 (-0.010, 0.007) 0.685 |

All covariates were adjusted in the multivariable regression model. P-values were calculated using weighted analyses. Bold values indicate statistical significance at P < 0.05.
